# Perceived effectiveness of complementary medicine by mothers of infants with colic in Gauteng

**DOI:** 10.4102/hsag.v24i0.1175

**Published:** 2019-02-26

**Authors:** Natalie C. Di Gaspero, Radmila Razlog, Reshma Patel, Janice Pellow

**Affiliations:** 1 Department of Homoeopathy, University of Johannesburg, South Africa

## Abstract

**Background:**

Infantile colic is a self-limiting condition, characterised by spasmodic, excessive and inconsolable crying without apparent cause. Although common, there is no widely accepted conventional treatment approach for colic. Complementary medicine is often promoted as an alternative therapeutic option for infantile colic; however, there is limited research available on its use, safety and effectiveness.

**Aim:**

The aim of this study was to determine the perceived effectiveness of complementary medicine by mothers of infants with colic by means of the Infantile Colic Questionnaire.

**Setting:**

Mothers of infants who had colic were recruited from complementary medicine pharmacies, schools, baby clinics and various businesses in Gauteng, South Africa.

**Methods:**

A quantitative-descriptive design was used whereby data was collected through a randomised, cross-sectional questionnaire. The research sample consisted of 152 participants (mothers), aged between 18 and 45 years, with one or more children who suffered from symptoms of infantile colic, who had used complementary medicine as a form of treatment.

**Results:**

Results indicated that most participants made use of both complementary and conventional medicines for their infant’s colic; the most commonly used complementary medicine products were homeopathic remedies, probiotics and herbal medicines. Some participants were, however, unfamiliar with the term ‘complementary medicine’, indicating a need for further patient education.

**Conclusions:**

The participants perceived complementary medicines as safe and effective forms of treatment for infantile colic. However, further, larger scale studies should be conducted to validate this finding.

## Introduction

Infantile colic is defined as a behavioural disorder that is characterised by spasmodic, excessive and inconsolable crying without apparent cause in an otherwise healthy infant. Infantile colic is a common but poorly understood condition that affects many infants between the ages of 2 and 16 weeks and is prevalent in both males and females. It is defined according to Wessel’s criteria as crying in a seemingly healthy infant that lasts for more than 3 h each day, on more than 3 days a week, for a period of more than 3 weeks (Savino et al. 2014b). Infantile colic occurs in 10% – 30% of infants, making it one of the most common reasons parents and their infants consult with medical health practitioners. Infantile colic is not only distressing to the infant but for the family, too, and because there is no widely accepted conventional treatment for colic parents may turn to complementary medical treatments (Savino & Tarasco 2010).

The aetiology of infantile colic is not fully understood despite its frequent occurrence. Research suggests that there may be numerous independent causes of this disorder (Savino et al. 2014b). One possibility stems from the notion of the gut hypothesis, with hypertonicity and increased formation of intraluminal gas resulting in infantile colic (Marek 2011). Another theory suggests that food allergies or hypersensitivities may be responsible for this condition. Other factors may also include lactose intolerance, hypermotility of the gut, gastro-oesophageal reflux and disruption in gut hormones or gut microflora (Savino & Tarasco 2010).

## Treatment approaches for infantile colic

The treatment of infantile colic is aimed at reducing the intensity of crying or eliminating factors that appear to exacerbate the crying. There is no gold standard treatment for colic and, as a result, many treatment options are utilised (Bailey, D’Auria & Haushalter 2013).

### Conventional medicine

There is a broad spectrum of both over-the-counter (OTC) and prescription medications available (National Cancer Institute 2016). In a review of treatments for infantile colic, it was found that there was little evidence to support many conventional medicines and that many of them are prohibited because of reported side effects. An integrative approach, combining both conventional and complementary medicines, has been found to be most effective (Bailey et al. 2013).

### Complementary medicine

Complementary medicine is defined by the World Health Organization (WHO) as ‘a broad set of health care practices that are neither part of that country’s own tradition, nor integrated into the dominant health care system’. In some countries, the term complementary medicine is often used interchangeably with ‘traditional or alternative medicine’ (a term that is used globally to describe traditional products, practitioners and practices) (WHO 2016). However, in South Africa, traditional medicine and complementary medicine are seen as two different modalities. According to the Medicines Control Council of South Africa, complementary medicine means any substance or mixture that originates from plants, minerals or animals that is intended to be used to alleviate or prevent illness (Medicines Control Council 2016). To guarantee the safety and efficacy of medicines, the South African Health Products Regulatory Authority controls their manufacture, distribution and sale (Medicines Control Council 2018).

Complementary medicines are often promoted in the treatment of infantile colic; however, there is limited research available on their use, safety and effectiveness in the treatment of this condition (Perry, Hunt & Ernst 2011). The majority of the medicines are currently unscheduled, allowing for them to be purchased over the counter without a prescription (Gqaleni et al. 2016).

There is a growing necessity and demand for complementary medicine in South Africa, despite conventional medicine being the main source of healthcare (Snyman 2014). This recent growth of the complementary medicine market in South Africa has resulted in complementary medicines being available in numerous retail outlets where they are obtainable without prior medical consultation (Gqaleni et al. 2016).

With limited research available on the use, safety and effectiveness of complementary medicines in the treatment of infantile colic, it is important to establish which complementary medicines are popular and perceived to be effective for infantile colic, allowing for further clinical, investigatory and safety research to be conducted on them (Perry et al. 2011). As such, the aim of this study was to determine the perceived effectiveness of complementary medicine by mothers of infants with colic, by means of the Infantile Colic Questionnaire.

The objectives for this study were as follows:

- to provide insight into the use and perceived effectiveness of complementary medicines
- to provide valuable information regarding the understanding of complementary medicine for this condition.

## Research methods and design

### Research design and procedure

A quantitative-descriptive design was used whereby data was collected through a randomised cross-sectional questionnaire.

### Research population and sample

The research sample consisted of 150 participants (mothers) recruited by means of purposive sampling. Inclusion criteria were mothers aged between 18 and 45 years, who had had one or more children with infantile colic, and who used complementary medicine as a form of treatment for the colic. The sample group of mothers were recruited from complementary pharmacies (*n* = 41), schools (*n* = 32), baby clinics (*n* = 54) and various businesses (*n* = 30) in Gauteng, South Africa, via word of mouth and advertising flyers.

### Reliability and validity measures

The Infantile Colic Questionnaire is a reliable tool used in research related to infantile colic and reflux, and was slightly modified to suit the objectives of this study (Hodge & Murphy 2014; Murphy 2015). Prior to the commencement of the study, five mothers who met the study criteria were asked to participate in a pilot study, pretesting the questionnaire. The results were not utilised in the analysis.

### Data collection and analysis

Participants were required to answer the questionnaire by crossing the appropriate response box or with a short, written response. This method allowed for ease of answering the questions and limited the time it took to complete the questionnaire, which encouraged greater participation in the study. Participants were provided with a private area in which to complete the questionnaire. The questionnaire took approximately 8–10 min to complete.

Statistical analysis was prepared with the assistance of Statkon using SPSS Statistics (Statistical Package for Social Sciences) (version 23). Quantitative data is presented as frequencies and custom tables. Multiple response analysis was conducted for questions where more than one answer could be selected, while answers to open-ended questions are reported (A. Kuhudzai, University of Johannesburg [STATKON], pers. comm., 13 December 2016).

### Ethical considerations

Ethical clearance was obtained from the University of Johannesburg Faculty of Health Sciences, Research Ethics Committee (REC-01-126-2016) and Higher Degrees Committee (HDC-01-46-2016) prior to conducting the research. The REC reports to the National Health Research Ethics Council of South Africa. There were no anticipated risks to the study. All participants were assured of privacy, confidentiality and anonymity and were informed of their right to withdraw from the study at any time, up until the questionnaire had been submitted, for whatever reason and without consequence.

## Results

A total of 220 questionnaires were printed and distributed to the various data collection areas in Gauteng. A total of 157 questionnaires were completed (response rate = 71.4%), of which only 152 questionnaires were utilised in the data collection as they were completed correctly with no omitted responses.

The mothers were aged 18–29 years (*n* = 49; 32.2%), 30–39 years (*n* = 67; 44.1%) and 40–45 years (*n* = 36; 23.7%) and had either one child (*n* = 62; 40.8%) or two (*n* = 60; 39.5%) children, both with and without colic. The majority of the infants were female (*n* = 156; 55.0%) and were diagnosed as having colic by a medical health practitioner (*n* = 116; 76.3%). The remainder of participants self-diagnosed their child’s colic. The results showed that the prevalent age for colic to start was between 4 and 8 weeks of age (*n* = 135; 88.8%), with the firstborn child being more likely to have suffered from colic (*n* = 104; 68.4%). Typically, only one child in the family experienced colic (*n* = 125; 82%). Results showed that most participants attended an antenatal class (*n* = 81; 53.0%) and were sufficiently taught how to take care of their newborns in the postnatal stage (*n* = 55; 67.9%).

Many participants reported that their infant experienced episodes of colic occurring twice a day (*n* = 49; 32.2%) or 3 days a week or more (*n* = 96; 63.1%). These bouts lasted around 11–20 min at a time (*n* = 48; 31.6%) with a total colic time of 22–40 min a day. A few participants reported that there were numerous times that they were unable to soothe their child.

In this study, the most common reasons stated for excessive crying in the infant were pain or discomfort (*n* = 127; 83.6%), being tired (*n* = 111; 73.0%) and a dirty nappy (*n* = 95; 62.5%). The least reported reasons were: the mothers’ tension (*n* = 37; 24.3%), the infant being fussy (*n* = 31; 20.4%) and the infant being nervous (*n* = 14; 9.2%).

Many mothers noted that colic symptoms occurred straight after feeding (*n* = 97; 63.8%) and that colic most often occurred in the evening (*n* = 61; 40.1%). The most common reasons for their infant being uncomfortable were build-up of wind (*n* = 114; 75.0%), difficulty bringing up wind (*n* = 90; 59.2%) and discomfort (*n* = 87; 57.2%). The least common reasons were food from the mother’s diet (*n* = 19; 12.5%), either food allergy or growth spurt (*n* = 15; 9.9%) and the environment (*n* = 14; 9.2%).

It was reported that (*n* = 108; 71.1%) of participants noted that their baby did vomit after being fed and that this was not a normal thing for them to do (*n* = 102; 67.1%).

Most mothers (*n* = 85; 55.9%) stated that it was difficult to determine the factor that induced sleep and they were not sure if it was because of the infant being comfortable and tired, or exhausted from crying.

Most of the participants used a combination of breastfeeding and bottle feeding (*n* = 68; 44.7%), and the feeding method was changed by (*n* = 98; 64.5%) of participants in order to try and ease the colic symptoms. Around (*n* = 51; 36.6%) of the participants exclusively breastfed and (*n* = 7; 4.6%) bottle-fed breast milk. In this study, it was reported that (*n* = 108; 71.1%) of participants fed their infants on demand from birth and they increased feeding intervals in the evening between 17:00 and 00:00 (*n* = 83; 54.6%). The majority of participants (*n* = 119; 78.3%) consciously burped their infants.

The use of a dummy and/or their thumb was reported by (*n* = 108; 71.1%) of participants as commonly used to self-soothe. The most effective methods of soothing reported by participants were bouncing or jiggling (*n* = 90; 59.2%), pacing (*n* = 78; 51.3%) and baby massage (*n* = 68; 44.7%), with the least effective being giving the infant warm water (*n* = 29; 19.1%), white noise (*n* = 14; 9.2%) and giving the infant sugar water (*n* = 14; 9.2%).

The use of both complementary medicine and conventional medicine to treat their infant’s colic was indicated by 73.0% (*n* = 111) of participants, while complementary medicine alone was only used by (*n* = 41; 27.0%) of participants.

The majority of participants (*n* = 104; 68.4%) had consulted with a complementary medicine practitioner. Participants gained their information regarding complementary medicine for colic mainly from complementary practitioners (*n* = 94; 61.8%), friends and family (*n* = 82; 53.9%), general medical practitioners (*n* = 76; 50.0%) and the Internet (*n* = 75; 49.3%).

**FIGURE 1:**
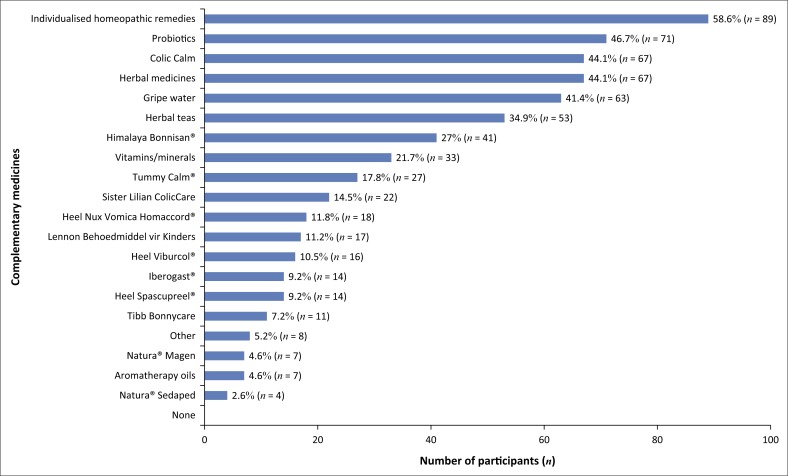
Complementary medicines used for colic.

Participants were also asked which complementary medicines they used for their child’s colic (Figure 1). More than one response could be selected. The most commonly used complementary medicines were as follows: (*n* = 89; 58.6%) individual homeopathic remedies; (*n* = 71; 46.7%) probiotics; (*n* = 67; 44.1%) either Colic Calm or herbal medicines; (*n* = 63; 41.4%) gripe water; (*n* = 53; 34.9%) herbal teas; (*n* = 41; 27.0%) Bonnisan^®^; and (*n* = 33; 21.7%) vitamins and minerals. Other complementary medicines that participants made use of that were not listed on the questionnaire were Bennetts^®^ colic mixture; Hylands colic tablets; Infacol and Neocate formula and Pegasus remedies, which collectively accounted for (*n* = 8; 5.2%) participants.

Participants were asked which conventional medicines, if any, they used to treat their infant’s colic. The most commonly used conventional medicines were Buscopan^®^ (*n* = 76; 50.0%); Telament Paediatric Colic Drops^®^ (*n* = 50; 32.9%); Muthi Wenyoni (*n* = 39; 25.7%); Colief^®^ Infant Drops (*n* = 34; 22.4%); and Nexium^®^ (*n* = 20; 13.2%). In addition, 3.3% (*n* = 5) of mothers used other products, namely Bennetts^®^ colic mixture and Lennon’s Behoedmiddel vir Kinders.

**FIGURE 2:**
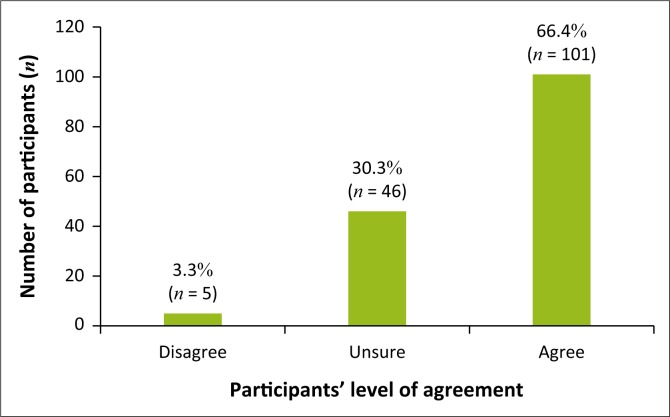
Perceived effectiveness of complementary medicine for colic.

It was found that (*n* = 101; 66.4%) of participants felt that complementary medicines were effective in treating colic, whereas (*n* = 46; 30.3%) were unsure and only (*n* = 5; 3.3%) did not believe it was effective (Figure 2).

**FIGURE 3:**
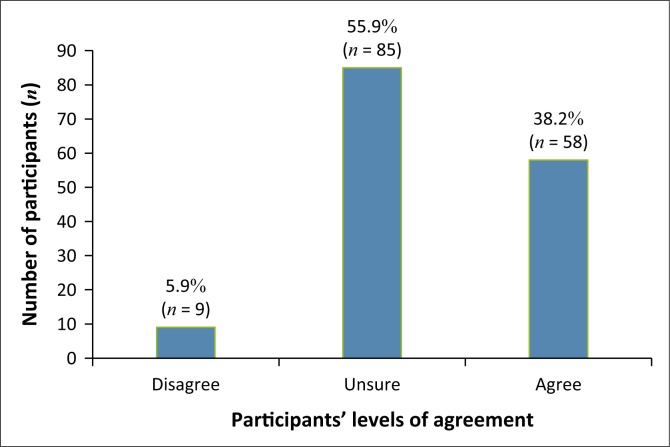
Perceived effectiveness of the combined use of conventional medicine with complementary medicine.

As seen in Figure 3, (*n* = 58; 38.2%) of participants felt that complementary medicine worked well in conjunction with conventional medicine, (*n* = 85; 55.9%) were unsure and (*n* = 9; 5.9%) disagreed.

**FIGURE 4:**
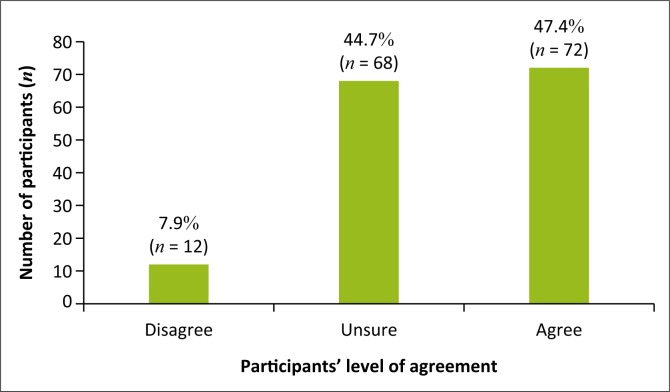
Did not experience side effects from complementary medicine use.

It was found that (*n* = 72; 47.4%) of participants agreed that in their experience complementary medicine had no side effects, (*n* = 68; 44.7%) were unsure and (*n* = 12; 7.9%) disagreed (Figure 4).

## Discussion

In this study, the majority of participants made use of both complementary and conventional medicines.

There is no widely accepted conventional treatment for colic and many parents feel dissatisfied with conventional healthcare, resulting in those parents seeking out complementary medicine treatments (Savino & Tarasco 2010). In a systematic review on randomised clinical trials on nutritional supplements and other complementary medicines for infantile colic, it was found that there was significant evidence for the effectiveness of fennel extract, mixed herbal teas and sugar solutions for infantile colic. Of the 15 included randomised clinical trials, 11 studies showed significant results in favour of complementary medicine. Yet despite the favourable outcome of complementary medicine for infantile colic, flaws in the included trials resulted in inadequate conclusions regarding the use of complementary medicine for infantile colic. The authors of this review therefore recommended that further research on the efficacy and safety of these complementary medicines be conducted (Perry et al. 2011).

It was also observed that the majority of participants had consulted with a complementary medicine practitioner. This finding could be directly related to the fact that three complementary (natural) pharmacies were utilised for data collection compared to the one conventional (mainstream) pharmacy. Doing so may have resulted in a high probability of the participants having been referred to a natural pharmacy by a complementary medicine practitioner. Moreover, because most participants had consulted with a complementary medicine practitioner, this could account for the high use of individualised homeopathic remedies. The majority of complementary medicines are currently unscheduled, allowing for them to be purchased over the counter without a prescription, resulting in an increase in self-medication (Gqaleni et al. 2016). Parents are advised to use herbal products with care or only through prescription by a registered complementary medicine practitioner in the relevant field (Savino & Tarasco 2010). The following products were utilised by the majority of participants: individualised homeopathic remedies, probiotics, either Colic Calm or herbal medicines, and gripe water.

Homeopaths prescribe individualised homeopathic treatment by taking into account all aspects of the infant and disease picture, thus providing a holistic individualised treatment (Vermeulen 2011). Over-the-counter homeopathic medicines, however, consist of combinations of remedies designed to relieve a variety of colic-related symptoms. According to Savino and Tarasco (2010), parents choose homeopathic remedies to relieve colic symptoms because of their non-toxic nature and very low concentrations of active ingredients.

The use of probiotics and herbal remedies has been found to reduce the median crying time in infants with colic (Bailey et al. 2013). In a recent randomised controlled trial by Martinelli et al. (2017) it was found that a mixture of *Matricariae chamomilla, Melissa officinalis* and *Lactobacillus acidophilus* or a mixture of *Lactobacillus reuteri* was significantly more effective in reducing colic symptoms (*p* < 0.001) than simethicone.

In a study by Chau et al. (2015), it was found that the probiotic *L. reuteri* was more effective than placebo in reducing the crying and fussing time in infants with colic. Savino and Tarassco (2010) found that breastfed infants who received a herbal formula containing *Matricariae recrutita, Foeniculum vulgare* and *M. officinalis* had a reduction in their colic symptoms within 1 week of use.

In this study, participants were also asked where they received their information regarding complementary medicine for colic. Most participants stated that it was recommended to them by a complementary medicine practitioner, a general medical practitioner or friends or family.

The use of complementary medicine continues to expand worldwide, with many people relying on them for primary healthcare. Herbal medicines, for instance, are viewed as natural products and patients assume that they are far safer and less likely to have side effects. However, this may not always be the case (Gqaleni et al. 2016) – particularly owing to the lack of standardisation of the dosage of herbal formulas and the possible content of sugar and alcohol in the formulas available (Savino & Tarasco 2010).

The most commonly used conventional medicines used by participants in this study were Buscopan^®^; Telament Paediatric Colic Drops^®^ and Muthi Wenyoni. Simethicone (Telament Paediatric Colic Drops), lactase (Colief^®^ Infant Drops) and hyoscine butylbromide (Buscopan) are commonly recommended OTC medications in South Africa for infantile colic.

Simethicone aims to reduce gas production. However, in several randomised placebo-controlled trials, simethicone was found to be ineffective (Bailey et al. 2013). Lactase is an effective form of treatment only in those cases where colic is a result of transient lactose intolerance (Savino et al. 2014b). A study conducted on the efficacy of hyoscine butylbromide found it to be an effective form of treatment for abdominal pain and cramping, and it is considered safe and well tolerated (Lacy et al. 2013). Muthi Wenyoni is an antacid consisting of calcium carbonate and magnesium carbonate, which helps to relieve dyspepsia by neutralising stomach acid (Resmed 2016). No clinical studies have been conducted on the efficacy of Muthi Wenyoni. It is suggested that the possible perceived effectiveness of Muthi Wenyoni is because of the alcohol content, which may induce sleep and relaxation in the infant (Bland et al. 2014). The unconvincing efficacy of conventional medicine products may be because of their potential side effects and unknown mode of action (Halpern & Coelho 2016). As such, pharmacists should inform customers that if the OTC colic preparation provides no relief for the colic after a few days, the product should be discontinued and they should consult with a doctor (Whittaker 2010).

In this study, participants were asked their opinions on statements regarding complementary medicine. More than half the participants agreed that complementary medicine is an effective form of treatment for infantile colic; this opinion is supported by Savino et al. (2014a), who state that complementary therapies have shown to be beneficial in infantile colic. Participants stated that they were uncertain if complementary medicine works well in conjunction with conventional medicine for infantile colic; this opinion is supported by Ben-Arye et al. (2010), as more clinical studies are needed on the integration of complementary and conventional medicine. Most participants agreed with the statement that complementary medicine had no side effects, and many were uncertain about the statement. The opinion that most participants felt that complementary medicine had no side effects is supported by Gqaleni et al. (2016), as the increase in the use of complementary medicine can be associated with its low frequency of adverse effects. Some participants were unfamiliar with the term ‘complementary medicine’.

## Limitations

There were a few factors that may have affected the results of the study:

- Numerous participants were unfamiliar with the term ‘complementary medicine’ and which OTC products belong to the complementary medicine category.
- A few participants made use of both complementary medicine and conventional medicine simultaneously, which made isolating the efficacy of the complementary medicine itself difficult.
- Participants who had more than one child with colic were conflicted when answering questions where the answer was different for each child. These cases resulted in a few questions having multiple answers.
- The age of the infants that were reported on was not recorded and this could therefore result in recall bias if an extended period of time had lapsed.

Future studies may offer potential improvement or refinement by incorporating the following recommendations:

- Conducting a similar study in other regions of the country will yield a more comprehensive perspective of the use of complementary medicine for colic in South Africa across different cultural groups.
- Providing definitions, synonyms and examples of complementary medicine and conventional medicine on the questionnaire will help participants have a better understanding of the terms.
- Including participants who have used complementary medicine as a singular form of treatment for colic could yield more conclusive results regarding perceived efficacy.
- The data collection tool should take into consideration participants who have had more than one infant suffering from colic.
- Future studies should be conducted to evaluate the efficacy and safety of available complementary medicines.

## Conclusions

Analysis of the results indicated that most participants made use of both complementary and conventional medicines for their infant’s colic. The medication was either used individually or simultaneously.

The most commonly used complementary products for infantile colic were individualised homeopathic remedies, probiotics and OTC herbal medicines. These results are consistent with other international studies conducted on integrative treatment approaches for infantile colic. Complementary medicine was chosen as a modality for treatment owing to it being perceived as safe, with fewer or no side effects. The most commonly used OTC conventional medications were products containing: hyoscine butylbromide, lactase and simethicone.

The results from this study indicated that participants perceived complementary medicine as an effective form of treatment for infantile colic. However, there is uncertainty regarding effectiveness as many parents used them in conjunction with conventional medicines.

Additional research and larger scale studies should be conducted to further validate this result.
